# Gene co-expression network analysis identifies BRCC3 as a key regulator in osteogenic differentiation of osteoblasts through a β-catenin signaling dependent pathway

**DOI:** 10.22038/IJBMS.2018.29498.7123

**Published:** 2019-02

**Authors:** Lixiong Cai, Zhiqian Huo, Haiyun Yang, Fengchun He, Zhenglin Cao, Feng Wu, Lianjun Liu, Bingyin Sun

**Affiliations:** 1 Department of Traumatology and Orthopedics, Foshan Hospital of TCM, Foshan 528000, China; 2 Department of Spine Osteopathy, Nanhai Hospital of Southen Medical University, Foshan 528000, China; 3 Department of Ophthalmology Myopia Treatment, Foshan Aier Eye Hospital, Foshan 528000, China; 4 Department of Orthopaedics, Foshan Jiangxiang Hospital, Foshan 528200, China

**Keywords:** BRCC3, β-catenin, Osteoblasts, Osteogenic differentiation, Osteoporosis

## Abstract

**Objective(s):**

The prognosis of osteoporosis is very poor, and it is very important to identify a biomarker for prevention of osteoporosis. In this study, we aimed to

identify candidate markers in osteoporosis and to investigate the role of candidate markers in osteogenic differentiation.

**Materials and Methods:**

Using Weighted Gene Co-Expression Network analysis, we identified three hub genes might associate with osteoporosis. The mRNA expression of hub genes in osteoblasts from osteoporosis patients or healthy donor was detected by qRT-PCR. Using siRNA and overexpression, we investigated the role of hub gene BRCC3 in osteogenic differentiation by alkaline phosphatase staining and Alizarin red staining. Moreover, the role of β-catenin signaling in the osteogenic differentiation was detected by using β-catenin signaling inhibitor XAV939.

**Results:**

We identified three hub genes that might associate with osteoporosis including BRCC3, UBE2N, and UBE2K. UBE2N mRNA and UBE2K mRNA were not changed in osteoblasts isolated from osteoporosis patients, compared with healthy donors, whereas BRCC3 mRNA was significantly increased. Depletion of BRCC3 promoted the activation of alkaline phosphatase and formation of calcified nodules in osteoblasts isolated from osteoporosis patients and up-regulated β-catenin expression. XAV939 reversed the BRCC3 siRNA-induced osteogenic differentiation. Additionally, inhibited osteogenic differentiation was also observed after BACC3 overexpression, and this was accompanied by decreased β-catenin expression.

**Conclusion:**

BRCC3 is an important regulator for osteogenic differentiation of osteoblasts through β-catenin signaling, and it might be a promising target for osteoporosis treatment.

## Introduction

Osteoporosis is a typical senile disease characterized by changes in bone microstructure, reduced trabecular bone density, thinning cortical bone, and fragile fracture (1). The increasing aging population of the world has led to osteoporosis, which has become the most prevalent and popular health problem, especially for women (2). This devastating disease causes an estimated 1.5 million fractures costing 16.9 billion dollars annually in the United States. It is estimated that osteoporotic fractures will increase by 48% to 3 million in 2025 and cost more than 25 billion dollars (3). Besides, only 25% of the patients with osteoporotic fracture can recover to pre-fracture functional levels (4). The prognosis of osteoporosis is very poor, which brings great burden to life and nursing. The gaining problem in China has become increasingly prominent, with increasing osteoporosis (5, 6). Thus, it is very important to identify a biomarker for prevention of osteoporosis (7).

Osteoporosis is mainly divided into primary, secondary, and idiopathic osteoporosis, of which secondary mainly includes endocrine, nutrition, drug, and genetics and so on. Postmenopausal osteoporosis belongs to primary osteoporosis (8, 9). Postmenopausal osteoporosis is associated with eating habits, heredity, and lifestyle habits, but the reduction of estrogen levels in postmenopausal women is the most important (10). Estrogen can promote osteogenic differentiation and osteogenesis of osteoblasts in many ways and play an important role in maintaining the balance between osteoblasts and osteoclasts (10-12). Estrogen plays an important regulatory role in bone growth, maturation, and metabolism. Postmenopausal increase in bone remodeling due to decreased levels of estrogen causes an imbalance between osteoblastic bone formation and osteoclastic bone resorption, which leads to a decrease in bone mineral density and an increased risk of fracture (11). Estrogen can promote osteoblast activity and has an anti-apoptotic effect on osteoblasts. Besides, estrogen can also inhibit osteoclast differentiation and maturation. Most of the functions of estrogen are achieved through estrogen receptor alpha and beta, both of these two receptors are widely expressed in bone marrow stromal cells and osteoblasts (13, 14). The level of estrogen is significantly reduced after menopause, the balance between osteoclasts and osteoblasts is broken, and bone resorption exceeds bone formation, leading to bone loss, and ultimately induces and accelerates the development of osteoporosis (15, 16). 

The Weighted Gene Co-Expression Network (WGCNA) is devoted to finding a co-expressed gene module and exploring the relationship between the gene module and the phenotypes concerned. As an efficient and accurate bioinformatics tool, the theory of WGCNA has been continuously developed and improved and has been widely used in biomedicine (17, 18). It has been successfully used for the identification of candidate markers in a variety of complex diseases including osteoporosis (19-21). Using mononuclear cell mRNA expression data from 12 low-bone-density and 14 high-bone-density premenopausal women detected by Dr. Leung Fung, Charles R Farber constructed the co-expression gene module and found that significant association exists between module 9 and bone mineral density (22).

In this study, we used WGCNA to identify the candidate markers in osteoporosis and reported an estrogen-independent molecular mechanism in differentiation and maturation of osteoclast isolated from osteoporosis patients.

## Materials and Methods

***Isolation of primary human osteoblasts and culture***

The primary human osteoblasts were achieved from the redundant trabecular bone fragments of osteoporosis patients and healthy donors. This study was approved by Ethics Review Board of Southern Medical University. All participants signed written informed consent. After minced into small pieces, the bone fragments were washed in PBS and digested with 2 mg/ml collagenase type II (300 U/mg, Sigma) for 2 hr at 37 ^°^C, and then placed in culture flasks with DMEM/F12 (Gibco, USA) containing 10% fetal bovine serum, 100 U/ml penicillin, 100 μg/ml streptomycin, and 1.25 μg/ml fungizone, and incubated at 37 ^°^C, 5%CO_2_. The culture mediums were changed twice a week. The primary human osteoblasts were confirmed by ALP staining. 

***Cell treatments***

The osteoblasts from osteoporosis patients were plated in 24-well plates (3×104 cells/well). For BRCC3 depletion studies, cells were transfected with NC or BRCC3 siRNA using Oligofectamine reagent (Invitrogen) according to the manufacturer’s instructions. The siRNAs

were synthesized by RiboBio Co, Ltd. (Guangzhou, China) and the sequences of BRCC3 siRNA were as follows: siRNA1-GCAGGAATTACAACAAGAA, siRNA2-GAAGGACCGAGTAGAAATT. After incubation for 24 hr, some cells were treated with XAV939 (5 mg/ml) to inhibit the β-catenin signaling. 

With osteoblasts from healthy donors, BRCC3 were overexpressed. At first, BRCC3 cDNA was prepared as previously described (23). In brief, the pCR3.1 TA mammalian expression vector was used to harbor the cDNA by cloning cDNA sequences between the restriction sites of BglII and Mlu1. After amplification and DNA sequence confirmation, cells received stable transfection with the vectors using Lipofectamine 2000 (Invitrogen, Carlsbad, CA) according to the manufacturer’s instructions. After a 6 hr transfection, the culture medium was replaced with the above mentioned for testing.

***Identification of osteoporosis-related genes***

We selected microarray data in GEO datasets GSE2208 (https://www.ncbi.nlm.nih.gov/geo/query/acc.cgi?acc=GSE2208) and GSE7429 (https://www.ncbi.nlm.nih.gov/geo/query/acc.cgi?acc=GSE7429). GSE2208 is the gene expression profile in 10 high bone mineral density (BMD) and 9 low BMD women. GSE7429 is the gene expression profile in 10 high BMD and 10 low BMD women. The shared genes were found out by Morpheus (https://software.broadinstitute.org/morpheus/) (24). Then, Gene Ontology (GO) term enrichment analysis and Kyoto Encyclopedia of Genes and Genomes (KEGG) pathway analysis for these shared genes were performed using DAVID version 6.7 (DAVID; david.ncifcrf.gov). *P*<0.05 was considered to be significantly different. Protein-protein interaction (PPI) network analysis was performed using the Cytoscape software package (ver. 3.4.0) with Molecular Complex Detection (MCODE) plug-in. When meeting the criteria of MCODE score ≥4 and number of node >4, the genes were selected as hub genes.

***qRT-PCR***

Total RNAs were isolated from osteoblasts using Trizol (Invitrogen, USA) according to the manufacturer’s instructions. Quantification of RNA was performed using a Nanodrop spectrophotometer (Nanodrop, USA). RNA was reversely transcribed with Bestar qPCR RT Kit (ABI, USA) with a PCR instrument (ABI9700, ABI, USA). Then, PCR was performed with DBI Bestar® SybrGreen qPCR Master Mix (ABI, USA) using a Real-time PCR system (Stratagene Mx3000P, Agilent, USA). The used primers are listed below: BRCC3 forward: 5′-GAACCCACTGCTTACTGGCTT-3′, reverse: 5′-TCGAGACCG AGGAGAGGGT-3′; UBE2N

forward: 5′-GGCAGCCCCTAAAGTACGTT-3′, reverse: 

5′-GCTTTATGCATGCTCAGGGC-3′; UBE2K forward: 

5′-CCGTCACAGGGGCTATTTGT-3′,reverse: 5′-GAATGGCCCTGACACACTCA -3′; GAPDH forward: 5′-CGGAGTCAACGGATTTGGTCGTAT-3′, reverse: 5′-AGCC-

TTCTCCATGGTGGTGAAGAC-3′. The PCR was performed for 2 min at 94 ^°^C, 40 cycles consisting of 20 sec at 94 ^°^C, 20 sec at 58 ^°^C, 20 sec at 72 ^°^C, and 1 min at 65 °C. The relative gene expression was calculated by the 2^−ΔΔCt^ method and GDPDH was used as the reference gene.

***Western blot***

Proteins were extracted from cells in RIPA buffer supplemented with protease inhibitors according to the standard procedure and quantified by BCA assay. 30 µg proteins were separated by 10% SDS-PAGE and blotted onto nitrocellulose membranes (Millipore, USA). Blots were blocked in 5% non-fat milk, incubated with primary antibody β-catenin (1:200) and GAPDH (1:1000) overnight at 4 ^°^C and horseradish peroxidase-conjugated secondary antibodies (1:5000) for 1 hr at 25 ^°^C. ECL reagent (Beyotime, China) was used for imaging.

***Alkaline phosphatase (ALP) staining***

After cells reached 80% confluence, the culture media was discarded. Cells were washed 3 times in PBS and fixed with 10% neutral formaldehyde for 20 min. The dyeing operation is referred to the alkaline phosphatase staining kit (Nanjing Jiancheng Bioengineering Institute, China) according to the manufacturer’s instruction.

***Alizarin red staining (ARS)***

After culturing for 21 days, the opaque area was found under the microscope. Cells were washed 3 times in PBS and fixed with 10% neutral formaldehyde for 20 min. Then, cells were stained with 0.1% alizarin red -Tris-HCl (pH 8.3) for 30 min and washed in tap water. Images were obtained using CX21 Olympus light microscopy (Olympus, Japan).

***Statistical analysis***

Each experiment was performed at least three times independently. Data are presented as mean ± standard error (SEM). Statistical analysis was performed using GraphPad Prism 5 (GraphPad Software, San Diego, CA, USA) using Student’s t-test. A *P*<0.05 was considered statistically significant.

## Results

***Identification of hub genes associated with osteoporosis***

**Figure 1:**
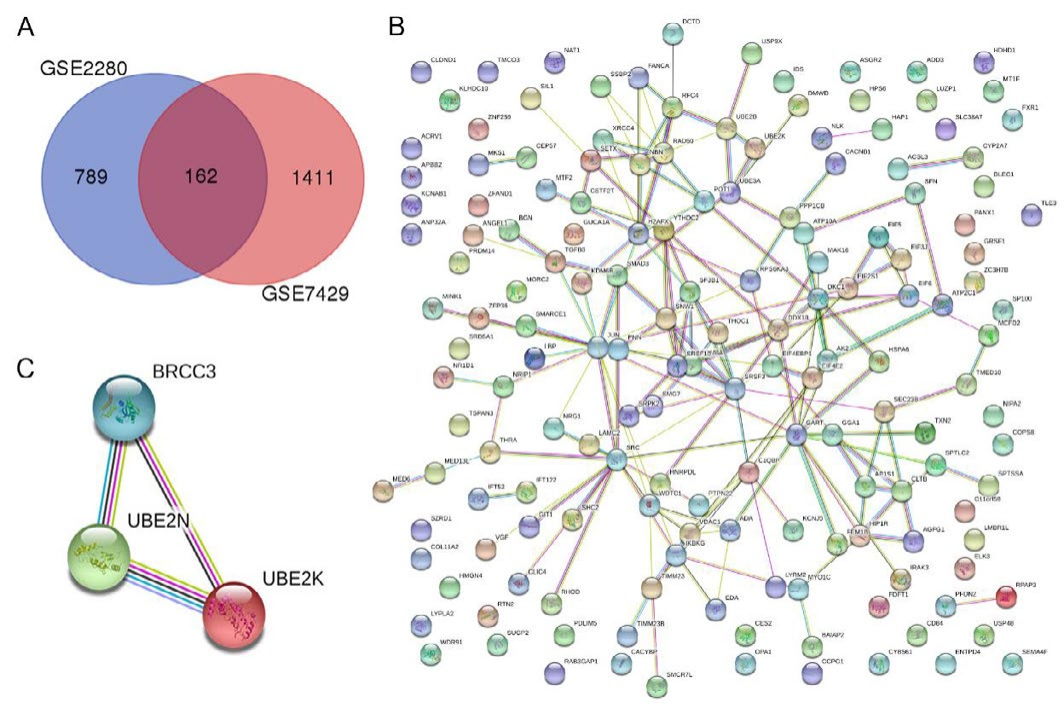
Identifying osteoporosis-related gene. (A) Wenn diagrams of differential expressed genes in GEO datasets GSE2208 and GSE7429; (B) PPI network among 162 common genes; (C) PPI network of hub genes BRCC3, UBE2N, and UBE2K

To identify the osteoporosis-related genes, we analyzed the differential expressed genes in GEO datasets GSE2208 and GSE7429. Results showed there were 162 common genes (Figure 1A). Further GO term enrichment analysis showed significantly enriched GO terms including apoptotic process, intracellular signal transduction, negative regulation of inflammation response, RNA binding, translation initiation factor activity, and negative regulation of macrophage cytokine production (Table 1). KEGG pathway analysis showed significantly enriched estrogen signaling pathway and prolactin signaling pathway (Table 1). Those GO term and KEGG pathways play important roles in osteoporosis (25-27). Thus, there were 52 genes that might be associated with osteoporosis (Table 1). PPI network analysis showed protein and protein interactions among the 162 common genes (Figure 1B). Then, three hub genes that have strong protein and protein interactions and might be associated with osteoporosis were identified, including BRCC3, UBE2N, and UBE2K (Figure 1C).

***BRCC3 up-regulated in osteoblasts from osteoporosis patients***

**Figure 2:**
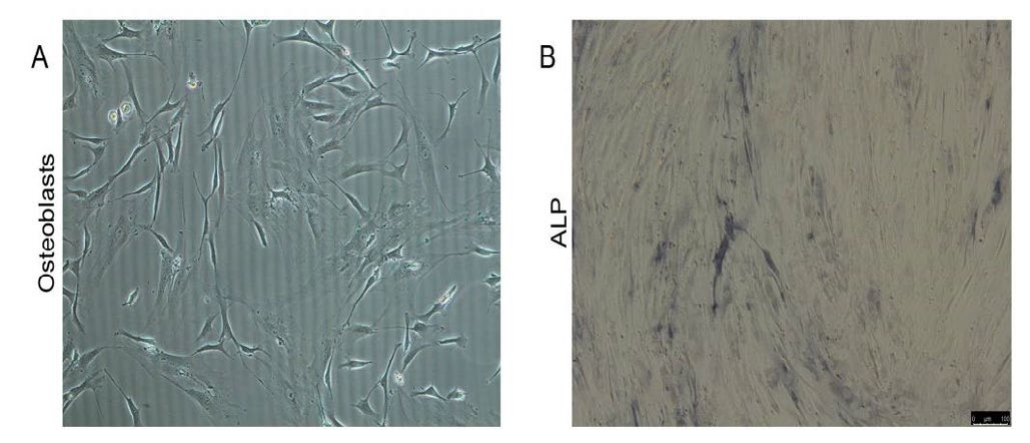
Identification of isolated osteoblasts. Primary osteoblasts were isolated. (A) Cell morphology; (B) ALP staining

Primary osteoblasts were isolated from osteoporosis patients and healthy donors. The cells have typical characteristics of osteoblasts that were fully extended, with shuttle, triangle, or irregular polygon morphology (Figure 2A). The osteoblasts were stained with ALP. Results showed brown or coffee granules in the cytoplasm, suggesting the isolated cells were osteoblasts (Figure 2B).

**Figure 3:**
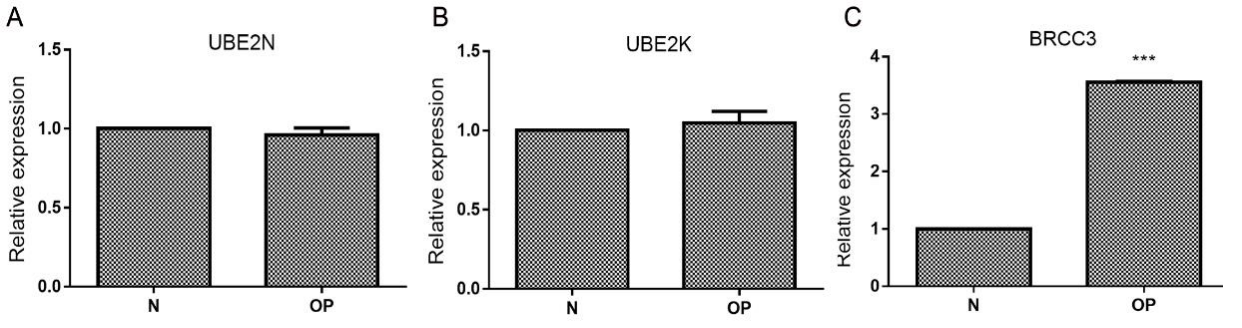
Levels of UBE2N, UBE2K, and BRCC3 in isolated osteoblasts. Primary osteoblasts were isolated from osteoporosis patients (OP) and healthy donors (N). (A) UBE2N mRNA; (B) UBE2K mRNA; (C) BRCC3 mRNA; ***P<0.001 vs N

The mRNA expressions of UBE2N (Figure 3A) and UBE2K (Figure 3B) not changed in osteoblasts from osteoporosis patients compared with healthy donors, while the mRNA expression of BRCC3 was significantly increased (Figure 3C).

***BRCC3 siRNA promoted differentiation of osteoblasts and up-regulated β-catenin***

**Figure 4:**
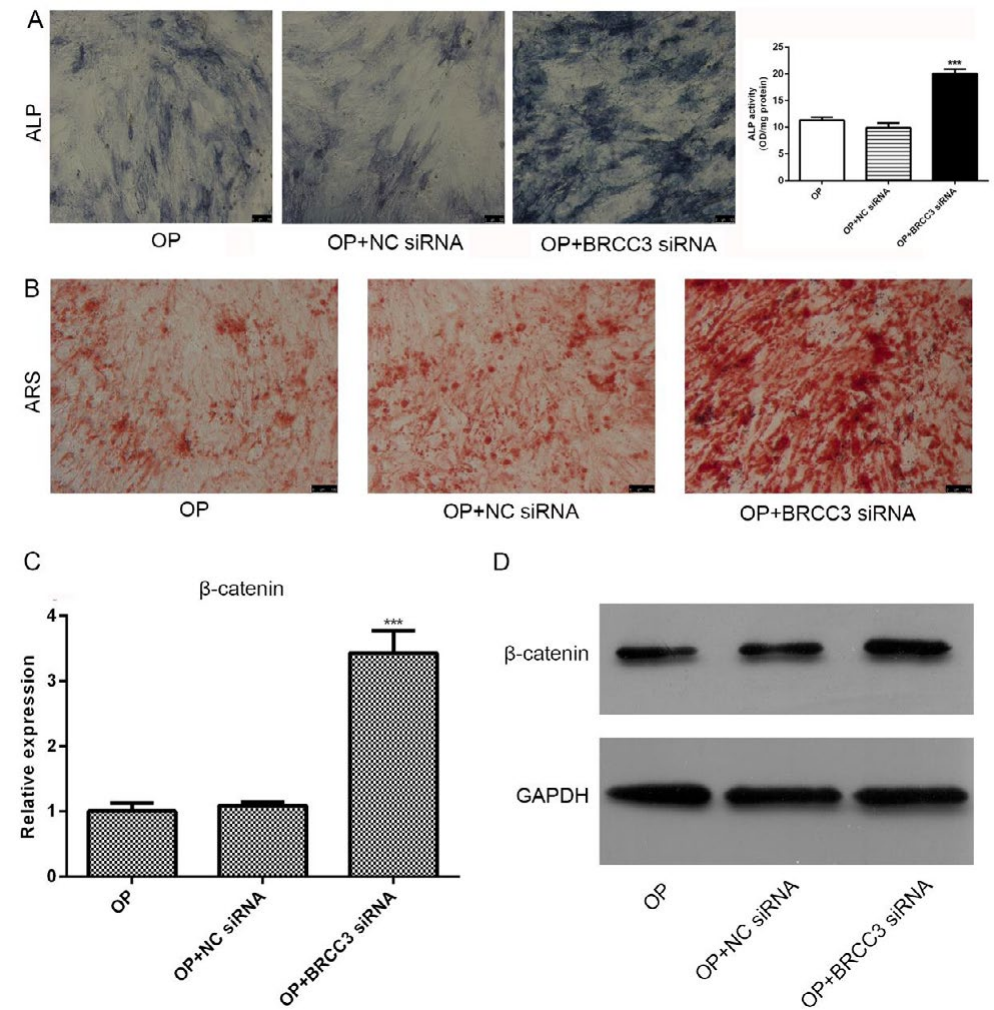
Effects of BRCC3 siRNA on differentiation of osteoblasts and expression of β-catenin. (A) ALP staining and quantitative analysis; (B) ARS; (C) β-catenin mRNA; (D) Western blot of β-catenin. ***P<0.001 vs OP

After transfected with BRCC3 siRNA and cultured for 21 days, the cells were stained with ALP and ARS (Figures 4A and B). Results showed that BRCC3 siRNA induced activation of ALP (Figure 4A) and promoted the formation of calcified nodules (Figure 4B). After transfection with BRCC3 siRNA, the expression of β-catenin was significantly increased in both mRNA and protein levels (Figures 4C and D).

***BRCC3 overexpression suppressed differentiation of osteoblasts and β-catenin expression***

**Figure 5:**
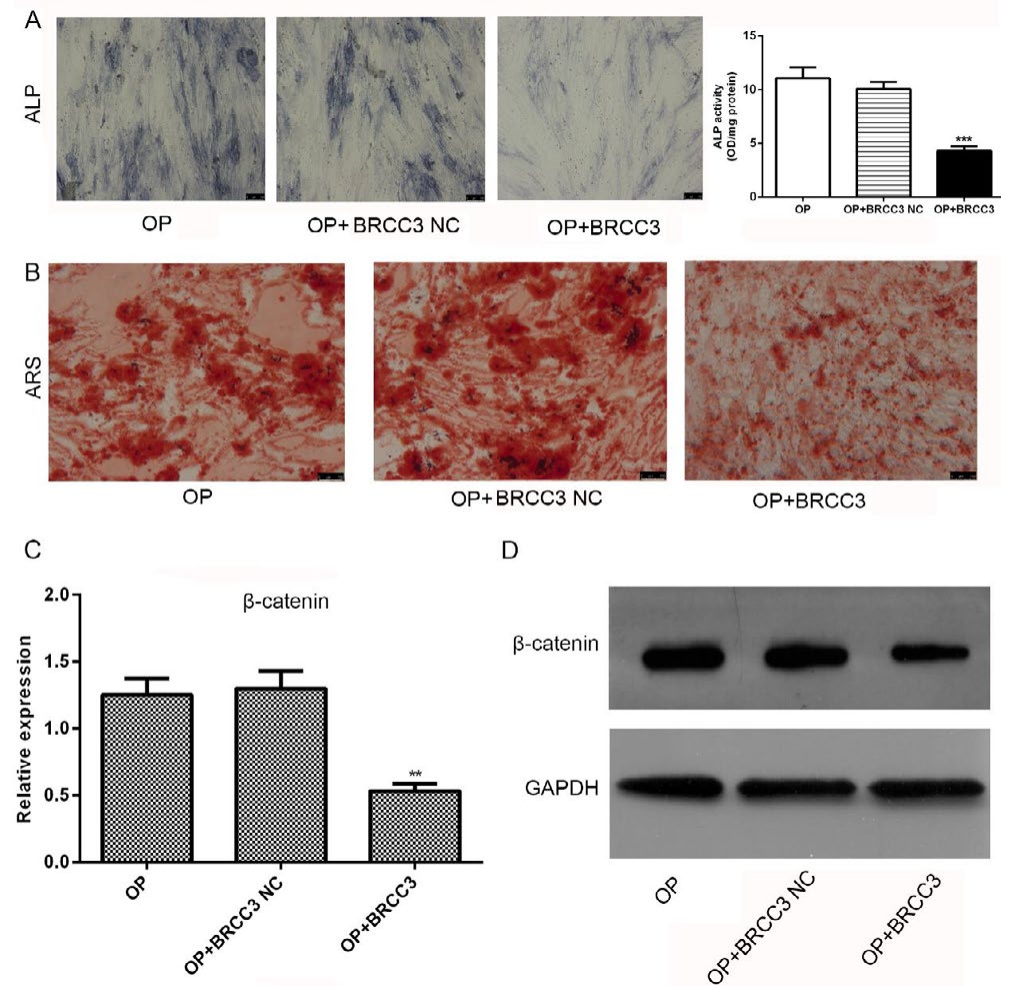
Effects of BRCC3 overexpression on differentiation of osteoblasts and β-catenin expression. (A) ALP staining; (B) ARS; (C) β-catenin mRNA; (D) Western blot of β-catenin. **P<0.01, ***P<0.001 vs OP

After transfection with BRCC3 cDNA and cultured for 21 days, the cells were stained with ALP and ARS (Figures 5A and B). Results showed that BRCC3 overexpression inhibited ALP activation (Figure 5A) and calcified nodules formation (Figure 4B). Additionally, BRCC3 overexpression significantly inhibited expressions of β-catenin at both mRNA and protein levels (Figures 5C and D).

***Inhibition of β-catenin reversed BRCC3 siRNA-induced osteogenic differentiation***

**Figure 6:**
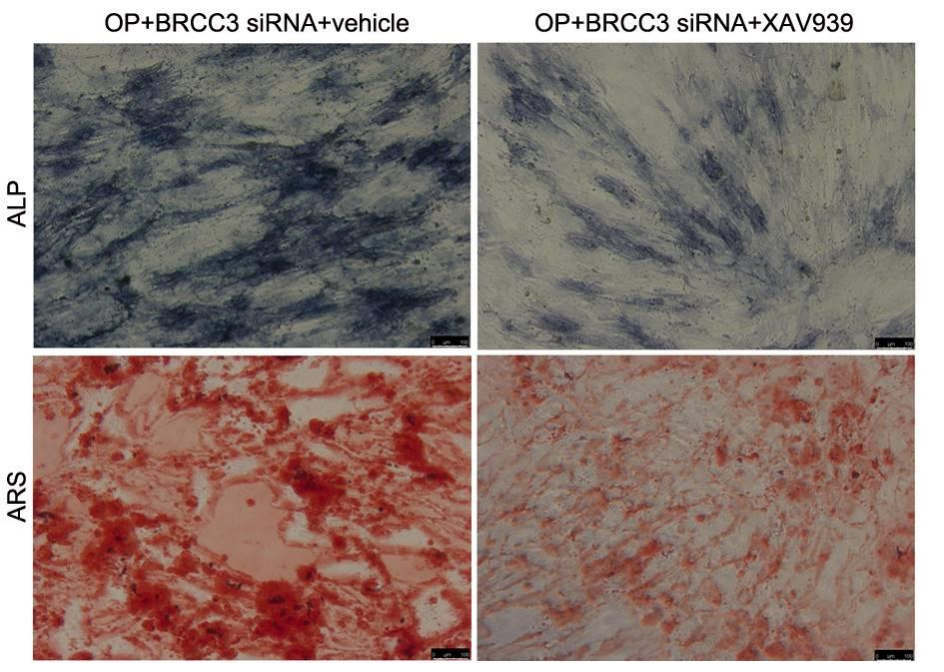
β-catenin signaling inhibitor XAV939 inhibited the BRCC3 siRNA-induced osteogenic differentiation. (A) ALP staining; (B) ARS

To test the role of β-catenin in BRCC3 siRNA actions, cells were treated with XAV939 to inhibit the activation of β-catenin signaling. Results showed that XAV939 inhibited the ALP activation and formation of calcified nodules induced by BRCC3 siRNA, suggesting that β-catenin signaling mediated the BRCC3 siRNA-induced osteogenic differentiation (Figure 6).

## Discussion

As a highly dynamic tissue, bone tissue is constantly undergoing reconstruction. Its balance is maintained by osteoblasts and osteoclasts (28, 29). Osteoclasts originate from monocyte macrophages in hematopoietic cells. Osteoblasts are derived from mesenchymal stem cells. Although these two cells are different in origin, they can regulate each other’s differentiation and function through interaction. Osteoblasts express two cytokines: macrophage colony-stimulating factor and receptor activator for the nuclear factor-κb ligand, all of which are necessary for osteoclastogenesis (30). Aging osteoporosis may be due to osteoblast growth disorders (31-33). In this study, we found BRCC3 mRNA and β-catenin mRNA were significantly increased in osteoblast isolated from osteoporosis patients. Moreover, depletion of BRCC3 promoted osteogenic differentiation of osteoblast through up-regulated β-catenin expression, whereas opposite effects were observed after BRCC3 overexpression.

**Table 1:** Enriched GO term and KEGG pathway associated with osteoporosis

Term	Description	Count	*P*-value
GO: 0006915	Apoptotic process	7	0.0009
GO: 00035556	Intracellular signal transduction	10	<0.0001
GO: 0050728	Negative regulation of inflammatory response	9	0.0010
GO: 0003723	RNA binding	8	0.0013
GO: 0003743	Translation initiation factor activity	5	0.0004
GO: 0010936	Negative regulation of macrophage cytokine production	5	0.0049
KEGG	Estrogen signaling pathway	4	0.0087
KEGG	Prolactin signaling pathway	4	0.0068

BRCC36 is a polypeptide encoded by the BRCC3 gene, containing 316 amino acids and a molecular weight of 36 kDa (34, 35). It was originally found that BRCC36 is a subunit of the BRCA1-BRCA2-containing complex (BRCC), which is also referred to as C6.1A, CXorf53, RP11-143H17.2. BRCC36 is a member of the JAMM / MPN + protease family that is characterized by its JAMM/MPN+ domain, which contains two histidine residues and one aspartic acid residues and can be stably bound to divalent zinc ions to form a catalytic center and thus plays an important role in deubiquitination (36). BRCC36 specifically recognizes ubiquitin chains formed by the linkage of ubiquitin lysine residues at K63 and hydrolyzes them to weaken the ubiquitylation (37). Studies have confirmed that ubiquitination mediated by K63 ubiquitin chain plays an important role in DNA damage repair, NF-κB signal transduction, cell cycle and ribosome function, and protein-targeted transport (38, 39).

BRCC36 functions intracellularly mainly through participation in the formation of both complexes of BRCC and BRISC (BRCC36 containing isopeptidase complex) (40); BRCC36 is involved in DNA damage repair (41). Researchers also found abnormally high expression of BRCC36 in tumor cells of patients with sporadic breast tumors that are characterized by tumor cells being less sensitive to radiotherapy, whereas depletion of BRCC36 results in increased sensitivity of cells to ionizing radiation (42). In this study, we found depletion of BRCC36 promoted the differentiation and activation of osteoblast, and β-catenin mediated this effect. BRCC36 may inhibit the activation of the TGF-β1 signaling pathway by regulating the ubiquitination of smad3 (43).

Osteoblasts differentiation is an extremely complex process involving the interaction of many cytokines, extracellular and extracellular matrix, as well as the cross-regulation of multiple signaling pathways (44). The enriched GO terms including apoptotic process, intracellular signal transduction, negative regulation of inflammation response, RNA binding, translation initiation factor activity and negative regulation of macrophage cytokine production, and enriched KEGG pathways including the estrogen signaling pathway and prolactin signaling pathway play important role in osteoporosis (25-27). It is generally believed that the Wnt/β-catenin signaling pathway and the TGF-β signaling pathway are involved in the regulation of osteoblast differentiation process (45). In the absence of Wnt signaling, β-catenin in the cytoplasm is mainly phosphorylated by the GSK-3β complex and then degraded by ubiquitination, thereby maintaining the low level of β-catenin in the cytoplasm and closing the pathway (46). Consistently, depletion of BRCC3 increased the level of β-catenin and promoted osteogenic differentiation, and inhibitor XAV939 inhibited the BRCC3-induced osteogenic differentiation. Additionally, the differentiation and β-catenin expression were suppressed by BRCC3 overexpression.

## Conclusion

In summary, BRCC3 is an important regulator for osteogenic differentiation of osteoblasts through β-catenin signaling, and it might be a promising target for osteoporosis treatment. However, the role of BRCC3 in osteogenic differentiation should be investigated by *in vivo* experiments.
